# A Comparative Study on Efficacy and Safety of Propofol versus Dexmedetomidine in Sleep Apnea Patients undergoing Drug-Induced Sleep Endoscopy:* A CONSORT-Prospective, Randomized, Controlled Clinical Trial*

**DOI:** 10.1155/2018/8696510

**Published:** 2018-11-01

**Authors:** L. L. Zhao, H. Liu, Y. Y. Zhang, J. Q. Wei, Y. Han, L. Han, J. P. Yang

**Affiliations:** ^1^Department of Anesthesiology, The First Affiliated Hospital of Soochow University, Soochow University, Suzhou 215006, Jiangsu, China; ^2^Department of Anesthesiology, The Affiliated Hospital of Xuzhou Medical University, Xuzhou 221004, Jiangsu, China

## Abstract

The aim of this study is to compare the efficacy and safety of propofol with dexmedetomidine in patients with obstructive sleep apnea hypopnea syndrome (SAHS) undergoing drug-induced sleep endoscopy (DISE). The 88 patients diagnosed with SAHS in the Affiliated Hospital of Xuzhou Medical University were randomly allocated into 2 groups (n = 44). Patients in the group dexmedetomidine (group D) received continuous intravenous infusion of dexmedetomidine 1 *μ*g/kg over 15 minutes before the endoscopy, and propofol 2 mg/kg was intravenously administrated in the group propofol (group P). Cardiopulmonary parameters of patients were recorded. The time to fall asleep, duration of endoscopic examination, the wakeup time of patients, the number of mask ventilations for patients, the satisfaction of patients and endoscopic performers, and false positive cases of SAHS of patients were compared between the two groups. Compared with group D, mean arterial pressure (MAP) and blood oxygen saturation (SPO_2_) of patients in the P group were lower at the time point of T1 (*P* < 0.05), the duration of endoscopic examination and wakeup time of patients were obviously prolonged, the incidence of mask ventilation for patients and false positive cases of SAHS of patients was observably higher, and the satisfaction of endoscopic performers was markedly lower, but the time to fall asleep was significantly shortened (*P* < 0.05). Dexmedetomidine served as a novel sleep induced drug and can provide satisfactory conditions and be safely and effectively applied for endoscopy in patients with SAHS, without adverse hemodynamic effects.

## 1. Introduction

Patients with SAHS have episodes of apnea or hypopnea due to the obstructive upper airways. Surgical operations are often considered as the alternative treatments in patients with SAHS. Therefore, to achieve site-specific surgical modification of the upper airway, it is vital to evaluate upper airway obstruction [1]. DISE can be used to observe the obstructive position and the anatomical factors causing local obstruction in the patients with SAHS under the condition of “sleep” and provide an important evidence for the reconstruction of the upper airway in these patients. A great number of studies have reported the validity and reliability of DISE. At present, the major drugs in clinical use for DISE are mainly confined to intravenous anesthetics and sedative hypnotic drugs, such as propofol, etomidate, and benzodiazepines [2]. Although studies showed that the target-controlled infusion of propofol can accurately control the depth of sedation during DISE, the ventilatory response to hypoxic can be depressed by larger doses of propofol during conscious sedation [3]. Furthermore, the sleep induced by all of these drugs used in the clinic for DISE is not in accordance with the natural physiological sleep, which interferes with the dynamic anatomical changes of airway causing apnea in the patients with SAHS.

Dexmedetomidine, a highly selective alpha 2 adrenergic receptor agonist, has a characteristic sedation similar to natural physiological sleep in conscious patients without significant respiratory depression [4, 5]. Therefore, dexmedetomidine is alternative drug used for conscious sedation in patients with SAHS undergoing DISE. However, the safety and efficacy of dexmedetomidine as a sedative used for DISE in the patients with SAHS are unknown.

Thus, in the present study, we compared efficacy and safety of dexmedetomidine with propofol for DISE in patients with SAHS and provided clinical evidences for dexmedetomidine as a novel sleep induced drug applied for endoscopy in patients with SAHS.

## 2. Material and Methods

### 2.1. Study Protocol

This prospective, randomized, controlled clinical trial was conducted at the Affiliated Hospital of Xuzhou Medical University, Xuzhou, Jiangsu, China, and the protocol of this study was approved by the Institutional Medical Ethics Committee of Xuzhou Medical University, in accordance with the approved guidelines. Written informed consent for participation in this study was obtained from all the participants before the start of the study. This trial is registered at the Chinese Clinical Trial Registry (ChiCTR-IOR-17010423). The sample size of the study was calculated according to the previous studies [6–9] and was based on a pilot study.

### 2.2. Patients

A group of 88 consecutive patients including 82 male and 6 female patients diagnosed with SAHS by the Department of Otorhinolaryngology, Head and Neck Surgery in the Affiliated Hospital of Xuzhou Medical University, aged between 33 and 60 years, were enrolled. Exclusion criteria included electrocardiogram suggesting bradycardia or cardiac conduction block, previous general anesthesia or recent administration of sedatives or analgesics, any known allergy to the study drugs, mental disease, nervous system disease, or developmental delay.

### 2.3. Randomization and Blinding

All patients were assigned by dint of a computer-generated random number table into one of two groups (n = 44): group D and group P. Drug-induced sleep was carried out by one experienced anesthetist, and the otolaryngologist was blinded to which kind of drug performed endoscopy.

### 2.4. Drug-Induced Sleep Endoscopy

All patients entered the examination room with quiet and dim light. The age, body mass index (BMI), apnea hypopnea index (AHI), and lowest oxygen saturation (LSaO2) of all patients in the two groups during polysomnography were recorded before drug-induced sleep endoscopy. All patients were not given any preoperative medication, with fasting for 6 hours before the examination, and lied on the examination couch for 10 minutes. Standard monitoring (MAP, SPO_2_, and ECG) was applied, and a peripheral intravenous cannula insertion was performed. 3-5 drops of ephedrine were given in the nasal cavity of patients for endoscopy. If the systolic pressure was higher than 180 mmHg or the diastolic pressure was above 110 mmHg, urapidil hydrochloride 5-10 mg was administrated. The anaesthetist monitored patients' vital signs and administrated drugs. Endoscopy was performed by the same otolaryngologist in this trial.

Patients in group D received continuous intravenous infusion of dexmedetomidine 1 *μ*g/kg (200 mg diluted in 50 ml saline) over 15 minutes before the endoscopy, and the maximum dosage of dexmedetomidine can be increased to 2 *μ*g/kg according to progress of endoscopic operation and the movement of the patients. Propofol 2 mg/kg was intravenously administrated in group P; then 1 mg/kg was given for maintenance, and the maximum dosage of propofol was 3 mg/kg. Endoscopy was performed by one otolaryngologist when patients disappeared the eyelash reflex. Before falling asleep, the electronic nasopharyngolaryngoscope was gently inserted and kept in the nasal cavity through checking the nasal cavity by the otolaryngologist, carefully holding the endoscopy until patient fell asleep. When the patient appeared to have respiratory effort or apnea, the degree of pharyngeal collapse of patients from the nasopharynx to larynx was recorded and saved as video for the later analysis.

The MAP of patients decreased ≥ 30% of the basic value, ephedrine 5-10 mg was intravenously injected, atropine 0.25-0.5 mg was infused when the HR was less than 50 bpm, and SpO_2_ of patients was lower than 50%; jaw was lifted to relieve obstruction and mask assisted ventilation with oxygen during endoscopy.

### 2.5. Outcome Measures

All related data were collected by another anaesthetist not directly involved in anaesthesia for the patient. The HR, SBP, DBP, and SpO_2_ of patients at time points of preadministration of drugs (T0), immediately falling asleep patients (T1), during endoscopic operation (T2), and completion of the endoscopy (T3) were recorded respectively. The time to fall asleep (T1-T0) was calculated. Duration of endoscopic examination, the wakeup time, number of mask assisted ventilations for patients, satisfaction of patients and endoscopic performers, and false positive cases of SAHS of patients in the two groups were recorded.

### 2.6. Postoperative Care

After completion of endoscopy, the drugs were taken off. After they were fully awake, patients continued to be carefully observed for 30 min and vital signs and SpO_2_ of patients were monitored, and the patients were allowed to be out of the examination room without any symptoms of dizziness, nausea, and vomiting.

### 2.7. Statistical Analysis

Data analyses first entailed characterization of participants using descriptive and summary statistics (mean ± SD for age, BMI, AHI, LSaO_2_, HR, MAP, SpO_2_, time to fall asleep, duration of endoscopic examination, and the wakeup time; percentages for sex ratio, mask assisted ventilation, the satisfaction of patients and endoscopic performers, and false positive cases of SAHS of patients). Data between two groups were compared using Student's* t*-test, or Pearson's chi-squared test. Statistical analyses of data were generated using Statistical Package for Social Science, version 11.0 (SPSS 11.0). All p values given are based on two-tailed tests and a* p* value of < 0.05 was considered statistically significant.

## 3. Results

**Figure 1:**
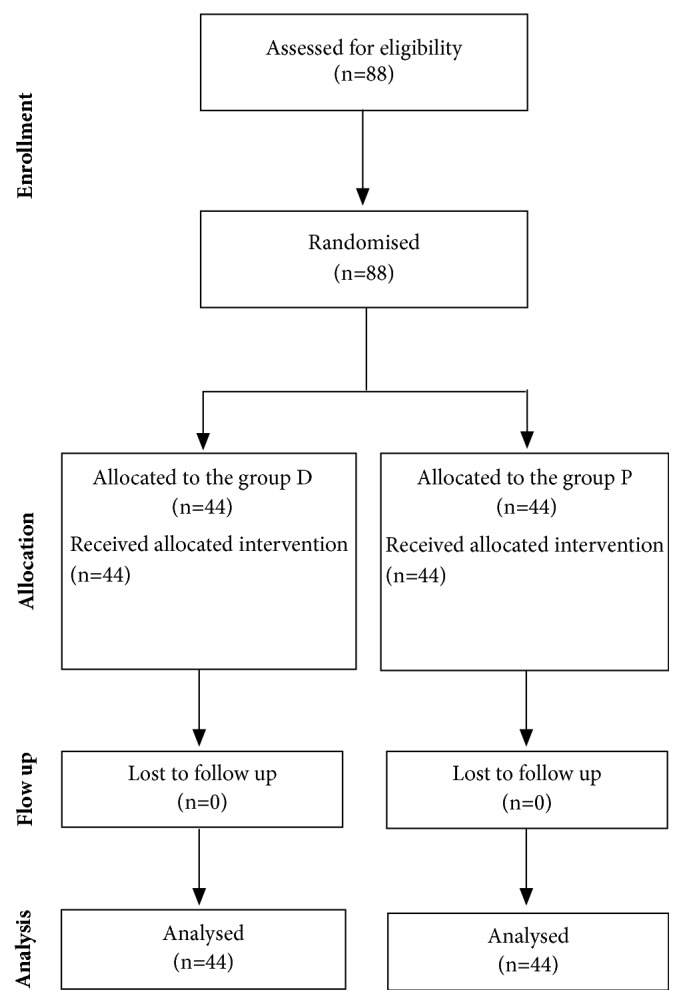
CONSORT flow diagram of patients included in the study.

Figure 1 shows the CONSORT flow diagram for patients in the present study. A total of 88 patients enrolled successfully completed DISE with propofol and dexmedetomidine in this study, and the data for all of these patients were analyzed.

Patient demographic data are shown in Table 1. There was no significant difference between the two groups in demographic data (age: 42.5 ± 6.0 in group D vs 43.2 ± 6.6 in group P; sex: 42/2 (male/female) in group D vs 40/4 (male/female) in group P), BMI (28.0 ± 3.5 in group D vs 28.9 ± 3.1 in group P), AHI (56.3 ± 21.5 in group D vs 54.3 ± 20.4 in group P), and LSaO_2_ (in group D vs 65.7 ± 0.21 65.8 ± 0.12 in group P).

Table 2 shows hemodynamic changes and SpO_2_ of patients in the two groups at time points of preadministration of drugs (T0), immediately falling asleep patients (T1), during endoscopic operation (T2), and completion of the endoscopy (T3). Both groups were comparable in values of baseline MAP, HR, and SpO_2_ at T0. Compared with group D, patients in group P displayed a significant decrease in MAP and SpO_2_ at T1 (*p* < 0.05). HR of patients in group D at T1-T3 and in group P at T1 was obviously lower than that of patients at T0 (*p* < 0.05). However, none of the patients required any drug treatment for hypotension or bradycardia.

The variables related to the efficacy and safety of the patients in both groups are shown in Table 3. Compared with group D, patients in group P displayed a significant prolongation in the duration of endoscopic examination and the wakeup time, an increase in the incidence of mask assisted ventilation for patients and false positive cases of SAHS, and decrease in satisfaction endoscopic performers; however, the time to fall asleep of patients in group P was significantly shorter (*p* < 0.05). None of patients in both groups required intubation due to significant respiratory depression after drugs administration, and even SaO2 < 50% in patients could be relieved by the mask assisted ventilation with oxygen.

## 4. Discussion

SAHS refers to the repeated apnea and (or) lower ventilation throughout sleep caused by a variety of reasons, characterized by a series of clinical symptoms such as repeated hypoxemia and disorder sleep structure during sleep, sleepiness and fatigue in daytime, and memory deterioration, which has serious harm to patient's health as a result of systematic pathophysiological changes induced by long-lasting chronic hypoxemia, hypercapnia, and disorder sleep structure [10–14]. Upper airway reconstruction as for the classical surgical treatment is the important technique to treat SAHS in the present clinic; however, the efficacy of nonselective surgery is not often ideal because of the lack of preoperative assessment of airway [15–17]. The fiberoptic nasopharyngoscope is commonly used to observe the morphological structure of the upper airway under direct vision and more likely show the dynamic changes of the upper airway during sleep. In 1991, Croft and Pringle et al. [18] first developed the DISE, where the condition of patient's upper airway collapse is dynamically observed using the fiberoptic nasopharyngoscope, under the condition of “sleep” induced by sedative medication.

However, over the past two decades the major drugs in clinical use for DISE were mainly confined to intravenous anesthetics and sedative hypnotic drugs, such as propofol, etomidate, and benzodiazepines [2]. But the sleep induced by all of these drugs used in the clinic for DISE is not in accordance with the natural sleep under the condition of physiology, which interferes with the dynamic anatomical changes of airway causing apnea. Therefore, it is necessary to find a new drug that can induce sleep without interference with the dynamic changes of the upper airway.

Dexmedetomidine, selectively binding to postsynaptic *α*_2_ adrenergic receptor located in the central nucleus of solitary tract, inhibits nervous impulse from sympathetic neurons in anterior horn of the spinal cord and reduces systemic sympathetic nervous tension and inhibits the release of norepinephrine from sympathetic nerve endings through activating presynaptic *α*_2_ adrenergic receptor, which produces sedative, analgesic, antianxiety, obstructive sympathetic and opioids spare [19–22]. In recent years, accumulating evidences suggest that dexmedetomidine can prevent postoperative nausea and vomiting, and shivering, and has protective effects for nerves, heart, and kidney through multiple mechanisms. Characteristic sedative effect of dexmedetomidine is similar to physiological sleep with awareness, which puts patients in the condition of sleep, and they can easily be awakened to communicate with medical examiners [23]. These features of dexmedetomidine make it a very commonly used drug in the early tracheal extubation and fast channel cardiac anesthesia, and it is safely used for patients requiring mechanical ventilation or spontaneous breath due to its less respiratory depression.

In this study, in order to provide clinical evidence of dexmedetomidine as a sleep-induced drug for the fiberoptic nasopharyngoscope, the feasibility and safety of dexmedetomidine used for the fiberoptic nasopharyngoscope were explored. We found that patients in group P had significant hemodynamic changes manifesting a decrease in MAP and SpO2 after infusion of propofol, which was associated with the side effects of propofol on different degrees of circular function inhibition and temporary respiratory depression. It increases potential risk of propofol as a sleep-induced drug for the endoscopy. However, although patients in group D had lower HR after administration, HR was still above 50 bpm, not requiring medication treatment, and there were no obvious hemodynamic change and respiratory inhibition. Further study showed that, compared with group D, although the time to fall asleep of patients in group P was significantly shorter, patients in group P displayed a significant prolongation in the duration of endoscopic examination and the wakeup time and an increase in the incidence of mask assisted ventilation for patients and false positive cases of SAHS, which decreased satisfaction of endoscopic performers.

Taken together, in the present study, we found that dexmedetomidine could satisfy the DISE requirement for patients undergoing SAHS and had no significant side effects on hemodynamic changes and less interference of breathing, which can be safely and effectively applied to DISE as the ideal induced sleep medication.

## Figures and Tables

**Table 1:** Demographics of analyzed patients.

	group D	group P
Age; years	42.5 ± 6.0	43.2 ± 6.6
BMI; kg/m^2^	28.0 ± 3.5	28.9 ± 3.1
Gender; male:female	42:2	40:4
AHI; events/hour	56.3 ± 21.5	54.3 ± 20.4
LSaO2; %	65.7 ± 0.21	65.8 ± 0.12

Data are presented as mean ± SD, or number of patients.

**Table 2:** Cardiopulmonary parameters of analyzed patients.

		T0	T1	T2	T3
MAP (mmHg)	Group D	109.7 ± 6.3	104.6 ± 13.9	101.1 ± 4.6	105.5 ± 7.3
	Group P	105.0 ± 13.8	89.4 ± 9.7^*∗*^	95.6 ± 9.3	104.3 ± 8.8
HR (bpm)	Group D	55.5 ± 4.3^#^	58.6 ± 5.2^#^	58.6 ± 5.2^#^	62.6 ± 7.4^#^
	Group P	69.0 ± 8.1^*∗*^	69.0 ± 8.1^*∗*^	75.9 ± 3.7	78.2 ± 5.4
SpO_2_ (%)	Group D	99.2 ± 9.2	98.7 ± 4.6	98.5 ± 7.3	99.5 ± 4.4
	Group P	99.5 ± 7.6	89.4 ± 5.7^*∗*^	92.4 ± 6.4	99.3 ± 5.6

Data are represented as a mean ± SD.

^*∗*^ *P* < 0.05 vs group D, and ^#^*P* < 0.05 vs T0.

**Table 3:** The variables related to the efficacy and safety of drug-induced sleep in analyzed patients.

	Preoperative administration of BP (n)	Time to fall asleep (min)	Duration of endoscopic examination (min)	Time to wakeup (min)	Mask ventilation (n)	Satisfaction of patients	Satisfaction of endoscopic performers	False positive cases of SAHS (n)
Group D	8	13.4 ± 2.5	20.2 ± 7.3	2.5 ± 0.7	0	98 ± 4.1	99 ± 2.7	4
Group P	9	6.6 ± 1.2^*∗*^	29.5 ± 4.6^*∗*^	5.7 ± 1.1^*∗*^	38^*∗*^	99 ± 1.7	90 ± 3.2^*∗*^	24^*∗*^

Data are represented as a mean ± SD.

^*∗*^ *P* < 0.05 vs group D.

## Data Availability

The data used to support the findings of this study are available from the corresponding author upon request.

## Abbreviations

AHI: Apnea hypopnea index
BMI: Body mass index
DISE: Drug-induced sleep endoscopy
HR: Heart rate
LSaO2: Lowest oxygen saturation
MAP: Mean arterial pressure
SAHS: Sleep apnea hypopnea syndrome.
